# Equivalence of sessile droplet dynamics under periodic and steady electric fields

**DOI:** 10.1038/s41526-021-00176-2

**Published:** 2021-11-16

**Authors:** Muhamed Ashfak Kainikkara, Dipin S. Pillai, Kirti Chandra Sahu

**Affiliations:** 1 Department of Chemical Engineering, Indian Institute of Technology Kanpur, Kanpur, Uttar Pradesh India; 2 Department of Chemical Engineering, Indian Institute of Technology Hyderabad, Sangareddy, Telangana India

**Keywords:** Chemical engineering, Mechanical engineering

## Abstract

The electrohydrodynamics of a sessile droplet under the influence of periodic and steady electric fields in microgravity conditions is theoretically investigated using an inertial lubrication model. Previous studies have revealed that a freely suspended spherical droplet with unequal conductivity and permittivity ratios exhibits distinct dynamics under periodic and equivalent steady forcing in the root mean-square sense. However, it is unclear when (if at all) such distinct dynamics occur for periodic and equivalent steady forcing in the case of sessile droplets. The equivalence between periodic and steady forcing is shown to be governed by the interfacial charge buildup, which further depends on the competition between the charge relaxation and forcing timescales. A circulation-deformation map is introduced for the sessile droplet that acts as a guideline to achieve electric field-induced wetting or dewetting as the case may be. We also demonstrate that a droplet may be rendered either more or less wetting solely by tuning the forcing frequency.

## Introduction

The dynamics of sessile droplets under the influence of electric fields has fascinated researchers for decades due to their importance in a variety of microgravity applications^1,2^ as well as technological operations ranging from electrostatic spraying, ink-jet printing, medical diagnostics to microelectronics^3,4^. Further, the effect of electric field on the shape of a sessile droplet is also a promising means to determine the surface tension of fluids under reduced gravity, where conventional techniques are not applicable^3,5,6^. Despite significant progress in the field over the years, there are still considerable gaps in our understanding of the parities and disparities of a droplet’s dynamical response under alternating (AC) and direct (DC) electrical fields, owing to the difficulties in capturing the underlying physics as well as the assumptions made about the electrohydrodynamic properties of the fluids. A comprehensive understanding of the effect of an electric field on droplets and bubbles is of importance both for terrestrial as well as space applications.

For leaky dielectric fluids, a few studies^7–9^ have shown, via two-dimensional numerical simulations, that the steady mean deformation of a droplet subject to an alternating electric field is equal to the steady-state deformation under an analogous root mean squared (RMS) direct electric field. However, for the case of a levitated droplet under an electric field, Torza et al.^10^ demonstrated experimentally that this is not true for all fluid-pair combinations. Recently, Sahu et al.^7^ also showed numerically that for a levitated spherical conducting droplet, the deformation behavior under the AC field may or may not always have the same mean amplitude as under the RMS DC field. They demonstrated that deformation behavior is influenced by the electrical conductivity (*σ*) and permittivity (*ε*) ratios between the droplet and the surrounding fluid. A concern that emerges then is what happens in the case of a sessile droplet under AC forcing for all possible combinations of timescales associated with charge relaxation and electric forcing, which can have significant ramifications in a variety of applications outlined above.

Many researchers have investigated the spreading of a sessile droplet on various surfaces under DC forcing, focusing on the transient spreading phase^4,11^ and the final equilibrium shape assuming a constant contact angle or pinned contact line^12^. Oh et al.^13^ used a combination of the dynamic contact angle model and the interfacial normal stress condition to study the electrowetting of a sessile droplet. Bateni et al.^6^ experimentally showed that in a steady electric field, polar sessile droplets increase their contact angle regardless of the field polarity, whereas for nonpolar droplets no influence on contact angle was observed. Mugele et al.^14^ found that a needle-like electrode undergoing attachment-detachment cycles with a droplet can cause it to oscillate, resulting in enhanced mixing. Later, they used AC electric forcing to produce oscillations in a sessile droplet to achieve chaotic mixing^15^. Lu et al.^16^ used molecular kinetic theory to describe the contact line dynamics of a sessile droplet under AC forcing and successfully match with their experimental results. The hydrodynamic flows inside a droplet under AC forcing were investigated by Lee et al.^17^. They observed that low-frequency flow is caused by shape oscillation in conjunction with contact line oscillation, whereas high-frequency flow is caused by the electrothermal effect, which is triggered by electrical charge generated due to electrical conductivity and permittivity contrasts across the interface.

The above review shows that the electrohydrodynamics (EHD) of a sessile droplet under AC electric forcing has received far less attention than under DC electric forcing, even though the former can expect substantially richer dynamics. Moreover, it is essential to establish whether the deformation seen under DC and AC forcing is analogous. Thus, we attempt to answer this question by performing a large number of numerical simulations for a wide range of electrical properties of the droplet fluid and employ a thin precursor film-based model developed using the weighted residual integral boundary layer (WRIBL) technique^18,19^. Our findings reveal that the mean droplet deformation under AC forcing is the same as corresponding RMS DC forcing when the timescale associated with charge buildup at the interface is small. The mean droplet deformation under AC forcing, on the other hand, deviates from that under equivalent RMS DC forcing when the relaxation timescale becomes comparable or greater than the forcing timescale. This phenomenon is explained via the distribution of surface charge formed at the interface and the associated flow field. The zero deformation curves for a sessile droplet under AC and DC forcing are also determined numerically in the electrical conductivity and permittivity ratios space.

## Methods

**Fig. 1:**
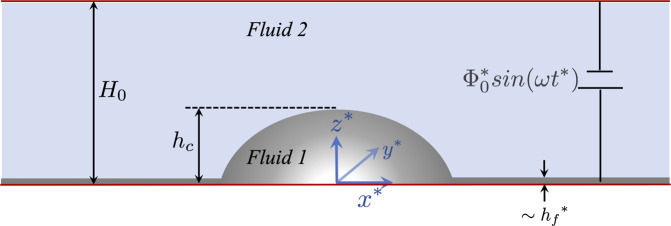
Schematic diagram. A three-dimensional sessile droplet (designated by *fluid 1*) subjected to a periodic electrostatic field between two planar electrodes separated by a distance *H*_0_. The droplet is hydrodynamically active, while the ambient fluid (designated by *fluid 2*) is assumed to be hydrodynamically passive. Note that the precursor film thickness is ${{{\mathcal{O}}}}({h}_{f}^{* })$^20^.

The schematic diagram shown in Fig. 1 displays a three-dimensional sessile droplet on a surface under the influence of an external electric field in a microgravity condition. The droplet (designated by *fluid 1*) is assumed to be a Newtonian fluid with density *ρ*_1_, and dynamic viscosity *μ*_1_, and the ambient fluid (designated by *fluid 2*) is taken to be hydrodynamically passive. The surface tension acting at the interface separating the fluids is denoted by *γ*. The electrical permittivity and conductivity of *fluids 1* and *2* are (*ε*_1_, *σ*_1_) and (*ε*_2_, *σ*_2_), respectively. A Cartesian coordinate system (x^*^, y^*^, z^*^) with its origin located at the center of the droplet on the bottom electrode is employed, such that u^*^, v^*^, and w^*^ denote the components of the velocity vector, ${\overrightarrow{v}}^{* }$ in the x^*^, y^*^, and z^*^ directions, respectively. Here, the asterisk is used to denote dimensional variables. In this study, we attempt to answer the following two questions: (i) is there an equivalence in the droplet deformation under AC and DC electric fields in the RMS sense?, (ii) if there is an equivalence, under what conditions does it exist? A parametric study is also conducted for a wide range of electrical permittivity and conductivity ratios to study the deformation of a sessile droplet under AC forcing.

A thin precursor film-based model^20^ is developed using the method of the weighted residual integral boundary layer (WRIBL). The advantage of the precursor film lies in the fact that the singularities associated with the triple contact line are conveniently avoided. The electrohydrodynamic behavior of the fluids is modeled using the leaky dielectric theory^21^. In the AC electric field, the bottom wall is held at an electric potential, ${{{\Phi }}}_{1}^{* }({z}^{* }=0)={{{\Phi }}}_{0}^{* }\sin (\omega {t}^{* })$, where ${{{\Phi }}}_{0}^{* }$ denotes the amplitude of the applied electric field, t^*^ denotes dimensional time, and *ω* represents the dimensional frequency of the applied AC electric forcing. On the other hand, in the equivalent DC case, ${{{\Phi }}}_{1}^{* }\,({z}^{* }=0)={{{\Phi }}}_{0}^{* }/\sqrt{2}$. In both cases, the upper electrode is grounded, i.e., ${{{\Phi }}}_{2}^{* }\,({z}^{* }={H}_{0})=0$. The governing equations and relevant boundary conditions are established in the following section.

### Governing equations

The electrohydrodynamics of a sessile droplet is governed by the continuity and the Navier–Stokes equations with the electrostatic Maxwell stress incorporated, which are given by

1 $$\nabla \cdot {\overrightarrow{v}}_{1}^{* }=0,\,\,\,{{\mbox{and}}}\,$$

2 $${\rho }_{1}\left(\frac{\partial {\overrightarrow{v}}_{1}^{* }}{\partial {t}^{* }}+{\overrightarrow{v}}_{1}^{* }\cdot \nabla {\overrightarrow{v}}_{1}^{* }\right)=\nabla \cdot {{{{\bf{T}}}}}_{1}^{* }+\nabla \cdot {{{{\bf{M}}}}}_{1}^{* }.$$

Here, the Cauchy stress tensor is given by ${{{{\bf{T}}}}}_{1}^{* }=-{p}_{1}^{* }{{{\bf{I}}}}+{\mu }_{1}({\nabla }^{* }{\overrightarrow{v}}_{1}^{* }+{\nabla }^{* }{\overrightarrow{v}}_{1}^{* T})$, where ${p}_{1}^{* }$ denotes the isotropic pressure field and **I** is the identity tensor. The Maxwell stress tensor (${{{{\bf{M}}}}}_{1}^{* }$) is the second invariant of the electric field gradient tensor that takes into account the stresses arising due to the applied electric field. This is given by

3 $${{{{\bf{M}}}}}_{1}^{* }={\varepsilon }_{0}{\varepsilon }_{1}\left[{\overrightarrow{E}}_{1}^{* }{\overrightarrow{E}}_{1}^{* }-\frac{1}{2}({\overrightarrow{E}}_{1}^{* }\cdot {\overrightarrow{E}}_{1}^{* }){{{\bf{I}}}}\right],$$

where ${\overrightarrow{E}}_{1}^{* }$ is the electric field in *fluid 1* and *ε*_0_ denotes the permittivity of free space. Under the leaky dielectric assumption, both fluids are assumed to be charge-free in the domain. Thus, ${\overrightarrow{E}}_{i}^{* }=-{\nabla }^{* }{{{\Phi }}}_{i}^{* }$ satisfies Gauss’s law, which for a charge-free domain reduces to:

4 $${\nabla }^{* 2}{{{\Phi }}}_{i}^{* }=0,$$

subject to the boundary conditions given by ${{{\Phi }}}_{1}^{* }={{{\Phi }}}_{0}^{* }\sin (\omega {t}^{* })$ at z^*^ = 0 and ${{{\Phi }}}_{2}^{* }=0$ at z^*^ = *H*_0_.

The interface speed (${{{{\mathcal{U}}}}}^{* }$) and the unit normal vector (${\overrightarrow{n}}^{* }$) are given by

5 $${{{{\mathcal{U}}}}}^{* }=\frac{\frac{\partial {h}^{* }}{\partial {t}^{* }}}{{\left[1+{\left(\frac{\partial {h}^{* }}{\partial {x}^{* }}\right)}^{2}+{\left(\frac{\partial {h}^{* }}{\partial {y}^{* }}\right)}^{2}\right]}^{1/2}},\,\,{{\mbox{and}}}\,\,{\overrightarrow{n}}^{* }=\frac{-\frac{\partial {h}^{* }}{\partial {x}^{* }}{\hat{i}}_{x}-\frac{\partial {h}^{* }}{\partial {y}^{* }}{\hat{i}}_{y}+{\hat{i}}_{z}}{{\left[1+{\left(\frac{\partial {h}^{* }}{\partial {x}^{* }}\right)}^{2}+{\left(\frac{\partial {h}^{* }}{\partial {y}^{* }}\right)}^{2}\right]}^{1/2}},$$

respectively. The interfacial tangent vectors (${\overrightarrow{t}}_{x}^{* }$) and (${\overrightarrow{t}}_{y}^{* }$) are given by

6 $${\overrightarrow{t}}_{x}^{* }=\frac{{\hat{i}}_{x}+\frac{\partial {h}^{* }}{\partial {x}^{* }}{\hat{i}}_{z}}{{\left[1+{\left(\frac{\partial {h}^{* }}{\partial {x}^{* }}\right)}^{2}+{\left(\frac{\partial {h}^{* }}{\partial {y}^{* }}\right)}^{2}\right]}^{1/2}},\,\,{{\mbox{and}}}\,\,{\overrightarrow{t}}_{y}^{* }=\frac{{\hat{i}}_{y}+\frac{\partial {h}^{* }}{\partial {y}^{* }}{\hat{i}}_{z}}{{\left[1+{\left(\frac{\partial {h}^{* }}{\partial {x}^{* }}\right)}^{2}+{\left(\frac{\partial {h}^{* }}{\partial {y}^{* }}\right)}^{2}\right]}^{1/2}},$$

where, ${\hat{i}}_{x}$, ${\hat{i}}_{y}$ and ${\hat{i}}_{z}$ are the unit vectors in the x^*^, y^*^, and z^*^ directions, respectively.

The kinematic condition at the material fluid interface (z^*^ = h^*^) is given as $\overrightarrow{{v}^{* }}\cdot {\overrightarrow{n}}^{* }-{{{{\mathcal{U}}}}}^{* }=0$, whence

7 $${w}^{* }=\frac{\partial {h}^{* }}{\partial {t}^{* }}+{u}^{* }\frac{\partial {h}^{* }}{\partial {x}^{* }}+{v}^{* }\frac{\partial {h}^{* }}{\partial {y}^{* }}.$$

At the interface, the tangential stress balance projected along the *x* − *z* and *y* − *z* planes and the normal stress balance are given by

8 $${\overrightarrow{n}}^{* }\cdot {{{{\bf{T}}}}}_{1}^{* }\cdot {\overrightarrow{t}}_{x}^{* }={Q}^{* }{\overrightarrow{E}}^{* }\cdot {\overrightarrow{t}}_{x}^{* },$$

9 $${\overrightarrow{n}}^{* }\cdot {{{{\bf{T}}}}}_{1}^{* }\cdot {\overrightarrow{t}}_{y}^{* }={Q}^{* }{\overrightarrow{E}}^{* }\cdot {\overrightarrow{t}}_{y}^{* },\,{{{\rm{and}}}}$$

10 $${\overrightarrow{n}}^{* }\cdot ({{{{\bf{T}}}}}_{1}^{* }+{{{{\bf{M}}}}}_{1}^{* })\cdot {\overrightarrow{n}}^{* }-{\overrightarrow{n}}^{* }\cdot {{{{\bf{M}}}}}_{2}^{* }\cdot {\overrightarrow{n}}^{* }=-\gamma {\nabla }_{s}^{* }\cdot {\overrightarrow{n}}^{* }+\frac{2s}{{h}_{f}^{* }}\left(\frac{{h}_{f}^{* 3}}{{h}^{* 3}}-\frac{{h}_{f}^{* 2}}{{h}^{* 2}}\right),$$

respectively^21^. Here, Q^*^ denotes the free charge density at the interface. In the tangential stress balance equations (Eqs. (8) and (9)), the right-hand side term corresponds to the shear stress arising as a consequence of the Coulomb force due to the presence of surface charges. As evident from Eq. (10), a jump in the normal component of the Maxwell stress between the two fluids at the interface results in additional stress. The last term in Eq. (10) is the conjoining-disjoining potential, with $s=\gamma \left(1-\cos \theta \right)$ being the wetting parameter that determines the equilibrium static contact angle (*θ*) of the droplet^20^. The thickness that minimizes the conjoining-disjoining potential is ${h}_{f}^{* }$, which sets the order of magnitude of the precursor film thickness. Gauss’s law and the continuity of the electric potential at the interface, z^*^ = h^*^ yields

11 $${\varepsilon }_{0}{\varepsilon }_{1}{\nabla }^{* }{{{\Phi }}}_{1}^{* }\cdot {\overrightarrow{n}}^{* }-{\varepsilon }_{0}{\varepsilon }_{2}{\nabla }^{* }{{{\Phi }}}_{2}^{* }\cdot {\overrightarrow{n}}^{* }={Q}^{* }\,{{{\rm{and}}}}\,{{{\Phi }}}_{i}^{* }={{{\Phi }}}_{2}^{* }.$$

The subtleties involved in the derivation of conservation equations for a surface field, specifically in the context of thin-film models, were elucidated by Pereira and Kalliadasis^22^. The equation governing interfacial charge dynamics is obtained from the charge conservation on a differential surface of the fluid interface^21^ as

12 $$\begin{array}{l}\left(\frac{\partial {Q}^{* }}{\partial {t}^{* }}+{\nabla }_{s}^{* }\cdot \left({Q}^{* }{\overrightarrow{v}}_{1s}^{* }\right)+{Q}^{* }\left({\nabla }_{s}^{* }\cdot {\overrightarrow{n}}^{* }\right)\left({\overrightarrow{v}}_{1}^{* }\cdot {\overrightarrow{n}}^{* }\right)\right)\\={\sigma }_{1}{\overrightarrow{E}}_{1}^{* }\cdot {\overrightarrow{n}}^{* }-{\sigma }_{2}{\overrightarrow{E}}_{2}^{* }\cdot {\overrightarrow{n}}^{* },\end{array}$$

where ${\overrightarrow{v}}_{1s}^{* }$ denotes the surface velocity and is given by ${\overrightarrow{v}}_{1s}^{* }={\overrightarrow{v}}_{1}^{* }-({\overrightarrow{v}}_{1}^{* }\cdot {\overrightarrow{n}}^{* }){\overrightarrow{n}}^{* }$. The surface gradient operator, ${\nabla }_{s}^{* }={\nabla }^{* }-({\overrightarrow{n}}^{* }\cdot {\nabla }^{* }){\overrightarrow{n}}^{* }$. The left-hand side of Eq. (12) corresponds to the material derivative of surface charge density as one moves along the surface, while the right-hand side represents the net rate of charge influx from the fluid bulk to the interface.

The above governing equations and boundary conditions are simplified using the thin-film approximation. The characteristic scales used for non-dimensionalization are as follows:

13 $$\begin{array}{l}{x}^{* }={{\Lambda }}x,\ \ {y}^{* }={{\Lambda }}y,\ \ {z}^{* }=Hz,\ \ {u}^{* }=Uu,\ \ {v}^{* }=Uv,\ \ {w}^{* }=(\delta U)w,\ \ \\{h}^{* }=Hh,\ \ {h}_{f}^{* }=H{h}_{f},\ \ {t}^{* }=({{\Lambda }}/U)t, {p}^{* }=({\mu }_{1}U/H)p,\ \ {{{\Phi }}}_{i}^{* }={{{\Phi }}}_{0}{{{\Phi }}}_{i},\ \\\ {Q}^{* }=({\varepsilon }_{0}{\varepsilon }_{2}{{{\Phi }}}_{0}/H)Q.\end{array}$$

Here, Λ is the characteristic length scale in *x*, *y* directions, which is representative of the wetted diameter of the droplet. The equilibrium height of the droplet in the absence of an electric field, *H*, is used as the length scale in the *z*-direction, such that *δ*( ≡ *H*/Λ) ≪ 1 (thin-film approximation). A characteristic velocity scale, *U* = *δ*^2^*γ*/*μ* is used, which is obtained by requiring the Capillary number, $Ca={\mu }_{1}U/\gamma ={{{\mathcal{O}}}}({\delta }^{2})$. In other words, the fluids considered here (e.g., water, lubricating oils, or liquid metal conductors) correspond to the large surface tension limit. Although the thin-film model employed in this study is only expected to be strictly valid for highly wetting droplets, numerical simulation has shown that it can accurately predict droplet dynamics for contact angles up to 30^∘^^23,24^. In all simulations, the nondimensional height of the top electrode, *β* = *H*_0_/*H*, is set to 3 such that *H*_0_ ≪ Λ. The dimensionless length of the computational domain, *L*, in the *x* and *y* directions are assumed to be the same, which is set to 2*L* = 8, with the droplet’s center located at (0, 0, 0). The complete derivation of the final evolution equations in the framework of the WRIBL technique is provided in the supplementary information. The final set of evolution equations for the nondimensional interface position (*h*), the depth-integrated flow rate in the *x*-direction (*q*_*x*_), and *y-*direction (*q*_*y*_), and the surface charge density (*Q*) are given by

14 $$\frac{\partial h}{\partial t}+\frac{\partial {q}_{x}}{\partial x}+\frac{\partial {q}_{y}}{\partial y}=0,$$

15 $$\begin{array}{l}\int\nolimits_{0}^{h}\delta ReF\left(\right.\frac{\partial \hat{u}}{\partial t}+\hat{u}\frac{\partial \hat{u}}{\partial x}+\hat{v}\frac{\partial \hat{u}}{\partial y}+\hat{w}\frac{\partial \hat{u}}{\partial z}\left)\right.dz\\={q}_{x}-\delta E{M}^{{T}_{1}}F{| }_{h}+\left[\right.\frac{{\delta }^{3}}{Ca}\frac{\partial }{\partial x}\left(\right.\frac{{\partial }^{2}h}{\partial {x}^{2}}+\frac{{\partial }^{2}h}{\partial {y}^{2}}\left)\right.\\\qquad\qquad\qquad\qquad\qquad\qquad\qquad\; -\delta E{M}^{{N}_{1}}+S\frac{\partial }{\partial x}\left(\right.\frac{{h}_{f}^{3}}{{h}^{3}}-\frac{{h}_{f}^{2}}{{h}^{2}}\left)\right.\left]\right.\int\nolimits_{0}^{h}Fdz,\end{array}$$

16 $$\begin{array}{l}\int\nolimits_{0}^{h}\delta ReF\left(\right.\frac{\partial \hat{v}}{\partial t}+\hat{u}\frac{\partial \hat{v}}{\partial x}+\hat{v}\frac{\partial \hat{v}}{\partial y}+\hat{w}\frac{\partial \hat{v}}{\partial z}\left)\right.dz\\={q}_{y}-\delta E{M}^{{T}_{2}}F{| }_{h}+\left[\right.\frac{{\delta }^{3}}{Ca}\frac{\partial }{\partial y}\left(\right.\frac{{\partial }^{2}h}{\partial {x}^{2}}+\frac{{\partial }^{2}h}{\partial {y}^{2}}\left)\right.\\\qquad\qquad\qquad\qquad\qquad\qquad\qquad\; -\delta E{M}^{{N}_{2}}+S\frac{\partial }{\partial y}\left(\right.\frac{{h}_{f}^{3}}{{h}^{3}}-\frac{{h}_{f}^{2}}{{h}^{2}}\left)\right.\left]\right.\int\nolimits_{0}^{h}Fdz,\end{array}$$

17 $$\delta {O}_{c}\left(\frac{\partial Q}{\partial t}+\hat{u}\frac{\partial Q}{\partial x}+\hat{v}\frac{\partial Q}{\partial y}+Q\frac{\partial h}{\partial x}\frac{\partial \hat{u}}{\partial z}+Q\frac{\partial h}{\partial y}\frac{\partial \hat{v}}{\partial z}-Q\frac{\partial \hat{w}}{\partial z}\right)=\left(\frac{\partial {\phi }_{2}}{\partial z}-\sigma \frac{\partial {\phi }_{1}}{\partial z}\right),$$

where *F* is the weight function, and its expression is given in the supplementary information; $\hat{u}$, $\hat{v}$, and $\hat{w}$ represent the leading order velocities in *x*, *y*, and *z* directions, respectively. The terms (${M}^{{N}_{1}},\,{M}^{{N}_{2}}$) and (${M}^{{T}_{1}},\,{M}^{{T}_{2}}$) denote the dimensionless normal and tangential components of the Maxwell stress in the *x* and *y* momentum equations, respectively. The various dimensionless numbers are the Reynolds number, *Re* ( ≡ *Uρ*_1_*H*/*μ*_1_); the dimensionless number associated with Ohmic charge conduction, *O*_*c*_ ≡ *ε*_0_*ε*_2_*U*/*σ*_2_*H*; $E\,(\equiv {\varepsilon }_{0}{\varepsilon }_{2}{\phi }_{0}^{2}/{\mu }_{1}UH)$ that denotes the dimensionless strength of electric field; *S*( ≡ 2*s*/*μ*_1_Uh^*^) representing the dimensionless wetting parameter; *σ*( ≡ *σ*_1_/*σ*_2_) that denotes the electrical conductivity ratio. In addition, *ε*( ≡ *ε*_1_/*ε*_2_) represents the electric permittivity ratio. Note that the three-dimensional governing equations are integrated in the *z*-direction to obtain the set of equations (14)–(17). This governs the dynamics of a three-dimensional (3D) droplet and hence is referred to as the “3D model”. The dynamics of a two-dimensional (2D) droplet extending infinitely in the third dimension was investigated by Pillai et al.^9^ and is referred to as the 2D model. The set of equations (14)–(17) recovers the 2D model of Pillai et al.^9^ in the appropriate limit.

The governing evolution equations (14)–(17) are solved using the following initial conditions:

18 $$h(x,y,0)={h{}_{e}}_{q}(x,y),\,\,{q}_{x}(x,y,0)=0,\,\,{q}_{y}(x,y,0)=0,\,{{\mbox{and}}}\,\,\,Q(x,y,0)=0,$$

where ${h{}_{e}}_{q}(x,y)$ is the equilibrium shape of the sessile droplet in the absence of an electric field, which is obtained by performing a transient simulation with *E* = *Q* = 0 until a steady-state droplet configuration is achieved. The initial conditions and spatially periodic boundary conditions used to obtain ${h{}_{e}}_{q}(x,y)$ are given by:

19 $${q}_{x,in}(x,y)=0,\,{q}_{y,in}(x,y)=0,\,{h}_{in}(x,y)\\=\left\{\begin{array}{ll}(1-{x}^{2}-{y}^{2})+{h}_{f},\quad &\,{{\mbox{if}}}\,| {x}^{2}+{y}^{2}| \le 1\\ {h}_{f},\quad &\,{{\mbox{if}}}\,| {x}^{2}+{y}^{2}| \,>\, 1.\end{array}\right.$$

20 $${q}_{x}(-L,y)={q}_{x}(L,y),\,\,{q}_{y}(-L,y)={q}_{y}(L,y),\,\,h(=-L,y)=h(L,y),\,\,\frac{{\partial }^{n}h}{\partial {x}^{n}}(-L,y)=\frac{{\partial }^{n}h}{\partial {x}^{n}}(L,y),\,n=1,2.$$

21 $${q}_{x}(x,-L)={q}_{x}(x,L),\,\,{q}_{y}(x,-L)={q}_{y}(x,L),\,\,h(x,-L)=h(x,L),\,\,\frac{{\partial }^{n}h}{\partial {y}^{n}}(x,-L)=\frac{{\partial }^{n}h}{\partial {y}^{n}}(x,L),\,n=1,2.$$

The initial condition ensures that the droplet volume remains constant when the values of parameters such as the wetting parameter, *S*, are changed. The Fourier spectral collocation technique is used to assure high-order spatial resolution and that the periodic spatial boundary conditions are satisfied. The NDSolve, a Mathematica v.12.0 function, is used to perform time integration using adaptive time stepping. A grid convergence test is conducted for a typical set of parameters in Supplementary Fig. 1. The 3D simulations are performed for three choices of grid points (*N*_*x*_ × *N*_*y*_) in the Fourier spectral collocation technique, viz., (50 × 50), (60 × 60), and (70 × 60). As the mean error in droplet deformation between (60 × 60) and (70 × 60) was found to be less than 1%, we choose (60 × 60) grids for all 3D simulations.

### Reporting Summary

Further information on research design is available in the Nature Research Reporting Summary linked to this article.

## Results and discussion

### Equivalence

**Fig. 2:**
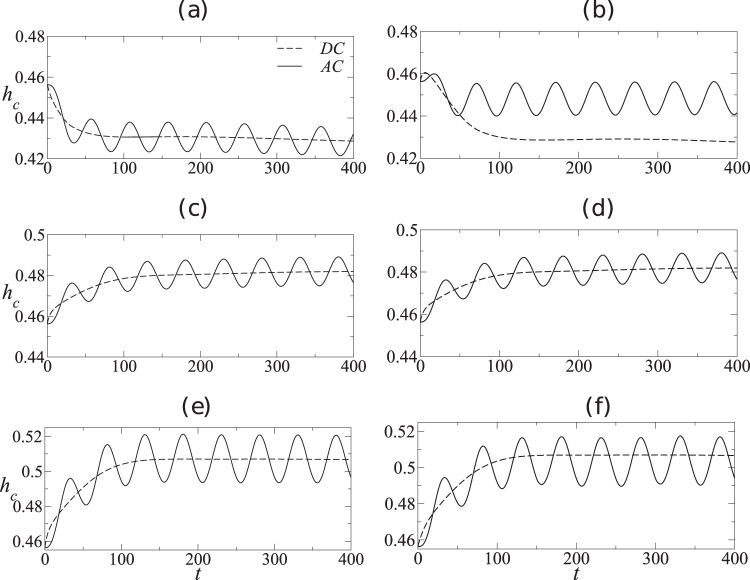
Comparison of droplet deformation between AC and equivalent DC fields. Temporal variation of droplet height, *h*_*c*_: (**a**, **c**, **e**) *O*_*c*_ = 0.01 and (**b**, **d**, **f**) *O*_*c*_ = 10. Panels (**a**, **b**), (**c**, **d**), and (**e**, **f**) are for (*ε* = 10 and *σ* = 6), (*ε* = 10 and *σ* = 10), and (*ε* = 6 and *σ* = 10), respectively. Here, for all the AC cases, Ω = *π*/50.

We begin the presentation of our results by answering the key question raised in this study, i.e., is there an equivalence between the mean deformation of a droplet under an AC field and its steady-state deformation under a corresponding RMS DC field? To answer this, in Fig. 2, we plot the temporal evolution of the centreline height of the droplet (*h*_*c*_) for both AC and equivalent RMS DC forcing. In all simulations, the following dimensionless parameters are held fixed, namely, *S* = 20, *E*_AC_ = 10, *E*_DC_ = 5, and *Re* = 1. The left panels (a, c, e) of Fig. 2 correspond to *O*_*c*_ = 0.01, while the right panels (b, d, f) correspond to *O*_*c*_ = 10. It is observed that when *O*_*c*_ is small (left panels), the mean droplet deformation under AC forcing remains the same as under equivalent RMS DC forcing. However, when *O*_*c*_ is large (right panels), the mean droplet deformation under AC forcing deviates from that under equivalent RMS DC forcing, as evident from panels 2b, f. Panel 2d however shows equal mean droplet deformation for both AC and equivalent RMS DC forcing. Thus, it can be concluded that the droplet deformation under AC forcing can, under certain conditions, be significantly different from the deformation under DC forcing. We now proceed to explain these observations based on the charge developed at the interface.

### Mechanism

The droplet shape deformation is a combined effect of the normal as well as the tangential Maxwell stresses acting on the interface. The normal Maxwell stress acting at the interface primarily depends on the disparity of electrical permittivity between the two phases. Thus, *ε* ≠ 1 is a necessary and sufficient condition for the existence of the normal Maxwell stress at the interface. The tangential Maxwell stress, on the other hand, is predominantly caused by the Coulombic force acting on the surface charges in response to the tangential component of the electric field. Thus, the presence of surface charges, as well as a tangential component of the electric field, is necessary for the tangential Maxwell stress to come into play. It should be noted that the sign and magnitude of the surface charge depend on the difference between conductivity and permittivity ratios in the system, i.e., (*σ* − *ε*)^25^.

**Fig. 3:**
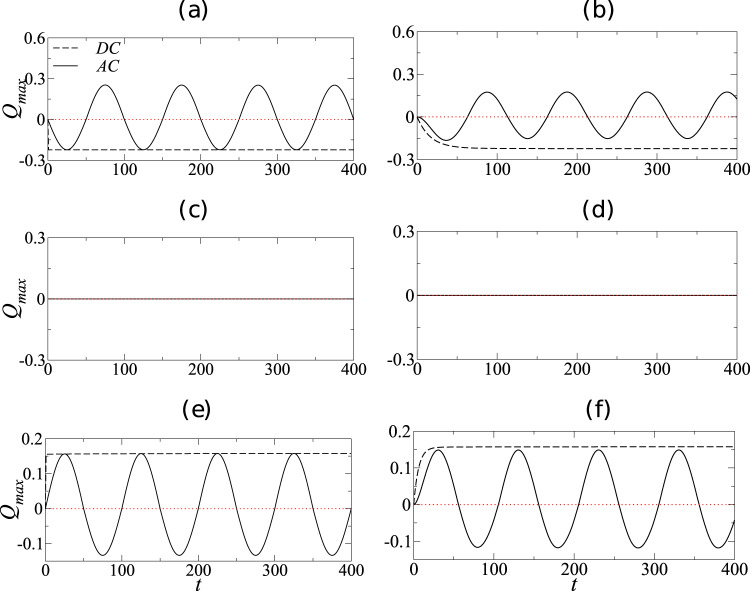
Interfacial charge buildup in the cases of AC and equivalent DC fields. Temporal variation of the maximum interfacial charge, *Q*_*max*_: **a**, **c**, **e** *O*_*c*_ = 0.01 and **b**, **d**, **f** *O*_*c*_ = 10. Panels (**a**, **b**), (**c**, **d**), and (**e**, **f**) are for (*ε* = 10 and *σ* = 6), (*ε* = 10 and *σ* = 10), and (*ε* = 6 and *σ* = 10), respectively. Here, for all the AC cases, Ω = *π*/50.

In Fig. 3, we present the corresponding temporal evolution of the maximum surface charge, *Q*_max_, for the same set of parameters used to obtain Fig. 2. We first discuss the DC results in each panel. The only relevant timescale for the evolution of surface charge in the DC case is the relaxation timescale, characterized by *O*_*c*_, which is the nondimensional timescale associated with charge buildup at the interface. When *O*_*c*_ is small (left panels), the timescale associated with charge buildup at the interface is small. This is evident from the left panels 3(a, e), where it can be seen that the surface charge for the DC case reaches its steady value quickly as compared to the right panels 3(b, d). Also, the sign and magnitude of the steady-state surface charge is governed by the term (*σ* − *ε*). Panels 3a, b correspond to *ε* = 10 and *σ* = 6, where *σ* < *ε* and therefore the steady-state charge is negative. As *ε* = *σ* = 10 in panels 3c, d, the steady-state value of charge is zero. Panels 3e, f are for *ε* = 6 and *σ* = 10, where *σ* > *ε* and therefore the steady-state value of charge is positive. Now for the AC case, there is an additional timescale corresponding to the periodic forcing, characterized by Ω, that influences the maximum attainable surface charge. It is in fact the competition between the forcing timescale and the relaxation timescale that determines whether the maximum surface charge under AC forcing can reach the equivalent DC steady-state value. It can be seen that when *O*_*c*_ is small (left panels in Fig. 3), the relaxation timescale is small and the maximum surface charge reaches its corresponding DC steady-state value. However, for large *O*_*c*_ (right panel in Fig. 3), the relaxation timescale becomes comparable to the forcing timescale and thus precludes the maximum surface charge from reaching its corresponding steady DC value. Thus, for higher values of *O*_*c*_, it is the difference in the magnitude of surface charge (and the resultant tangential Maxwell stress) between AC and DC cases that leads to a corresponding deviation in the *h*_*c*_ values. In panel 3d, as *σ* = *ε*, the surface charge is zero and the deformation is solely due to normal Maxwell stress, leading to similar AC and DC responses. With further increase in *O*_*c*_, i.e., *O*_*c*_ ≫ 1, the interface charge approaches zero and the system reaches a perfect dielectric limit.

As discussed in the introduction, for a freely suspended spherical droplet, Esmaeeli and Halim^8^ used a 2D Cartesian model and showed that the shape oscillations under AC electric field occur about the steady-state deformation observed under an equivalent RMS DC field. However, the experimental results of Torza et al.^10^ and axisymmetric simulations of Sahu et al.^7^ demonstrated that this is only true when the electrical conductivity ratio equals the permittivity ratio. This motivates us to investigate whether the deviation observed in the case of a sessile droplet (as shown in Fig. 2) arises due to the geometrical dimensionality. Thus, we have performed 2D simulations similar to that of Pillai et al.^9^ and compared with the corresponding 3D simulations. Figure 2 and Supplementary Fig. 2 present the results from 2D and 3D simulations respectively for the same set of parameters. It can be seen that our 3D model predicts qualitatively similar results as given by the 2D model for all parameters investigated. Comparing all six panels in Fig. 2 and Supplementary Fig. 2, it is evident that all AC and DC results obtained from the 2D model match qualitatively with the corresponding 3D model. Hence, a simpler 2D model proves to be extremely useful in providing physical insights into the problem, at a much lesser computational cost. We exploit this observation and employ the 2D model to obtain the rest of the results presented below.

**Fig. 4:**
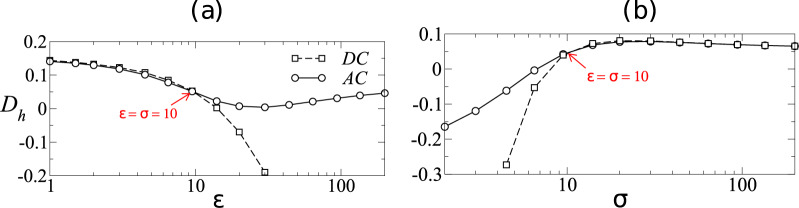
Deviation in droplet deformation between AC and DC fields as ***ε > σ***. Variation of fractional deviation of droplet height, *D*_*h*_ with **a** *ε* for *σ* = 10 and **b** *σ* for *ε* = 10. Here, *O*_*c*_ = 10 and for all the AC cases, Ω = *π*/50.

As discussed earlier, *O*_*c*_ = 10 corresponds to the case where competition between relaxation timescale and forcing timescale results in a disparity in the maximum surface charge between AC and DC cases. We now investigate more in detail this case of *O*_*c*_ = 10 to understand the effect of *ε* and *σ* on the deviation between AC and DC forcing. In Fig. 4a, we plot the droplet shape deformation, *D*_*h*_, as a function of *ε* for *σ* = 10. Here, *D*_*h*_ ≡ (*h*_*c*,*s*_ − *h*_*c*,0_)/*h*_*c*,0_, wherein *h*_*c*,0_ is the height of the droplet at *t* = 0 and *h*_*c*,*s*_ denotes the steady-state height of the droplet under DC forcing and the long-time steady mean height of the droplet under AC forcing. Similarly, Fig. 4b depicts the droplet shape deformation, *D*_*h*_, as a function of *σ* for *ε* = 10. It can be observed that when *σ* = *ϵ*, *D*_*h*_ for both AC and DC cases are equal. This is attributed to the absence of surface charge and consequently no tangential Maxwell stress at the interface. Further, the deviation of *D*_*h*_ between AC and DC is negligible when *σ* > *ε*. This is due to the fact that as *σ* increases, the fluid tends to approach the perfect conductor limit with more or less uniform electric potential in the fluid. Thus, even though the charge accumulated is proportional to (*σ* − *ε*), the tangential electric field is small due to weaker potential gradients in the fluid, and the resulting tangential Maxwell stress remains weak. When *σ* < *ε* and the difference between *σ* and *ε* increases, the resultant steady-state surface charge for DC case increases as it is proportional to (*σ* − *ε*). However, the maximum surface charge for the AC case is limited by the system *O*_*c*_, due to the competition of the two timescales (relaxation and forcing) discussed before. This, therefore, results in an increase in deviation in the *D*_*h*_ values between AC and DC.

### Circulation-deformation map

**Fig. 5:**
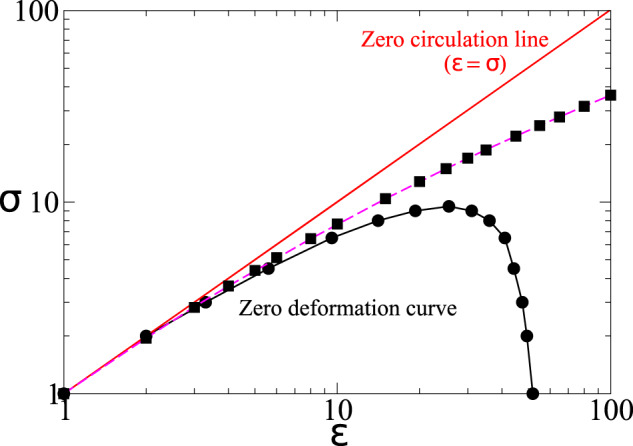
Circulation-deformation map. Zero circulation line and zero deformation curve in *ε* − *σ* space for *O*_*c*_ = 10 (filled circles) and *O*_*c*_ = 0.01 (filled squares) for AC electric forcing with Ω = *π*/50. The corresponding DC field result is shown by the dashed magenta line. Note that in the DC case, the zero deformation curve is independent of *O*_*c*_.

In the seminal work of Torza et al.^10^ on a spherical droplet placed in a time-periodic electric field, it was shown that the *ε* − *σ* parameter space can be divided into three regions by two curves corresponding to zero circulation and zero deformation. The zero circulation curve, given by *σ* = *ϵ*, corresponds to a zero surface charge. The direction of flow circulation inside the spherical droplet reverses as one crosses this curve in the *ε* − *σ* parameter space. In the absence of charge convection and fluid inertia, they also obtained an analytical expression for the zero deformation curve in the *ε* − *σ* parameter space, given by *σ*^2^ + *σ* + 1 − *ε* = 0. Above this curve, the spherical droplet deforms to a mean prolate shape, while below this curve, the droplet deforms to a mean oblate shape. A prolate configuration corresponds to a deformed droplet with its major axis in the direction of the field, while the major axis is in the direction perpendicular to the electric field for an oblate shape. These theoretical predictions of Torza et al.^10^ were confirmed by recent numerical investigations^7,8^, which showed that a spherical droplet exhibits markedly different behavior in the three regions of *ε* − *σ* parameter space, demarcated by the two curves. We now proceed to investigate the zero circulation and zero deformation curves for the sessile droplet. The zero circulation curve, which corresponds to zero surface charge is the same for the sessile droplet and is given by *σ* = *ϵ*. Also, an interesting observation from Fig. 4 is that *D*_*h*_ is found to be greater or less than zero, depending on the choice of *ε* and *σ*. This suggests the existence of a zero deformation curve in the *ε* − *σ* parameter space for the sessile droplet as well. It should be noted that the zero deformation curve is a consequence of the effect of normal Maxwell stress at the interface being nullified by an equal and opposite effect of the tangential Maxwell stress. While an analytical expression of the zero deformation curve was possible for a spherical droplet using spherical eigenfunctions as obtained by Torza et al.^10^, the same is not possible for a sessile droplet as the equilibrium shape itself is obtained numerically. Therefore, the zero deformation curve has to be obtained numerically via a series of computations. In Fig. 5, we plot the zero circulation and numerically obtained zero deformation curves for *O*_*c*_ = 10 (filled circles), *O*_*c*_ = 0.01 (filled squares), and equivalent RMS DC (dashed magenta line). As expected, the DC and *O*_*c*_ = 0.01 results coincide, in agreement with previous discussions of equivalence of DC for *O*_*c*_ = 0.01, as observed in Figs. 2 and 3. For *O*_*c*_ = 10, however, the zero deformation curve deviates from the equivalent DC case due to the deviation in charge buildup as discussed earlier. A further increase in *O*_*c*_ will cause the zero deformation curve to move further toward the bottom left, with the area under the curve shrinking. Finally, the curve will collapse to a single point (*ε* = *σ* = 1) in the limit of *O*_*c*_ ≫ 1, which corresponds to the limit of a perfect dielectric droplet with zero surface charge, due to the competition between the timescales associated with the charge relaxation at the interface and the electric forcing. Our calculations also revealed (but not shown here) that the zero deformation curve is only mildly sensitive to the parameter *S*, which determines the equilibrium contact angle.

**Fig. 6:**
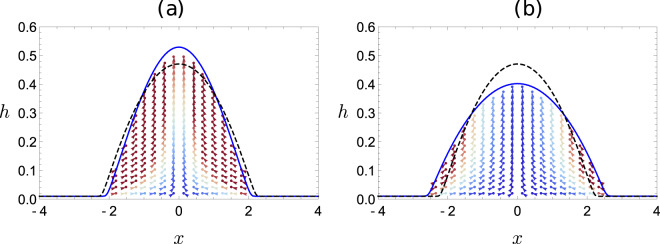
Internal flow profile. The velocity field inside the sessile droplet under AC field at *t* = 39.25*T*_*p*_. **a** *ε* = 2, *σ* = 20, and **b** *ε* = 10, *σ* = 2. The remaining parameters are Ω = *π*/50 and *O*_*c*_ = 10. The equilibrium shape of the droplet without an electric field (dashed curve) is used as the initial condition.

Above the zero deformation curve, *D*_*h*_ > 0, and the AC field renders the droplet less wetting, with the droplet occupying a lesser area on the substrate. Conversely, below this curve, *D*_*h*_ < 0, and the wettability increases with a larger droplet occupying a larger wetted area on the substrate. The droplet shape profile and the internal velocity field corresponding to the two regions is depicted in Fig. 6a, b for (*ε* = 2, *σ* = 20) and (*ε* = 10, *σ* = 2), respectively. The dashed curve in Fig. 6a, b is the equilibrium droplet shape in the absence of electric field, while the solid blue curve depicts the deformed droplet shape at a time instant corresponding to one-quarter of the forcing cycle (at *t* = 39.25*T*_*p*_, where *T*_*p*_ = 2*π*/Ω is the dimensionless time period of forcing field). As evident from Fig. 6a, the droplet is less wetting in the presence of AC field for (*ε* = 2, *σ* = 20), which lies above the zero deformation curve. Similarly, the droplet is more wetting for (*ε* = 10, *σ* = 2), which lies below the zero deformation curve. Further, the earlier discussions imply that the zero deformation curve will collapse to a single point (*ε* = *σ* = 1) for both perfect dielectric as well as a perfect conducting droplet due to the absence of tangential Maxwell stress in either case. Therefore, in both these limits, *D*_*h*_ > 0, the sessile droplet will always be rendered less wetting.

### Wetting transition

**Fig. 7:**
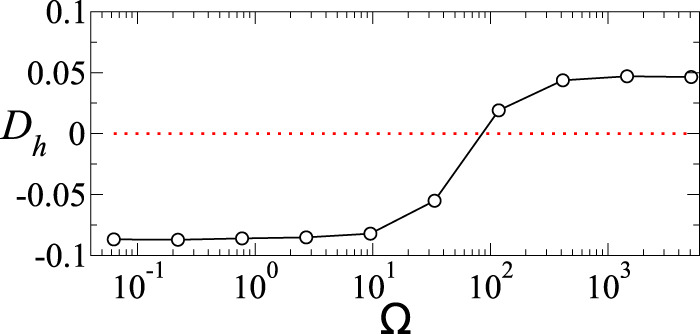
Wetting transition with forcing frequency. Variation of *D*_*h*_ versus Ω for *ε* = 10, *σ* = 6, and *O*_*c*_ = 0.01. The droplet undergoes a transition from more wetting (*D*_*h*_ < 0) to less wetting (*D*_*h*_ > 0) as forcing frequency is increased.

Based on the understanding of the competition between the two timescales under AC forcing, we now investigate the possibility to render a sessile droplet more wetting or less wetting solely by tuning the forcing frequency. In Fig. 7, the droplet deformation, *D*_*h*_, as a function of the forcing frequency, Ω, is plotted for *ε* = 10, *σ* = 6, and *O*_*c*_ = 10. For very low Ω, *D*_*h*_ < 0, and saturates to its equivalent DC steady-state value. With an increase in Ω, *D*_*h*_ increases and eventually turns positive. At very large Ω, the droplet approaches the perfect dielectric limit with zero interfacial charges, and hence *D*_*h*_ value saturates to the corresponding perfect dielectric case, independent of Ω.

To summarize, by conducting a large number of numerical simulations in the framework of a thin precursor film-based modeling, we show that the equivalence of sessile droplet dynamics under AC and corresponding RMS DC forcing depends on the competition between the two timescales, viz., the charge relaxation timescale, *O*_*c*_, and the forcing timescale, Ω. The origin of the deviation in droplet dynamics between DC and AC forcing is shown to arise due to a disparity in the interfacial charge buildup. Based on these understandings, a circulation-deformation map for the sessile droplet in the *ε* − *σ* parameter space is presented. Finally, we show the possibility of employing AC forcing to render a sessile droplet more wetting or less wetting, solely by changing the forcing frequency.

## Supplementary information

Reporting Summary

Supplementary Information

## Data Availability

The datasets generated during and/or analysed during the current study are available from the corresponding author on reasonable request.

## Code availability

The custom code used in the study are available from the corresponding author on reasonable request.
