# Supplementary material for: Equivalence of sessile droplet dynamics under periodic and steady electric fields
Source: NPJ Microgravity. 2021 Nov 16;7:47. doi: 10.1038/s41526-021-00176-2 (PMC8595882; doi:10.1038/s41526-021-00176-2)
Supplement: Supplementary file 2 — Supplementary Information [file 41526_2021_176_MOESM2_ESM.pdf]

Supplementary Information
Equivalence of sessile droplet dynamics under periodic and steady electric fields

Muhamed Ashfak Kainikkara[1], Dipin S. Pillai[1a] and Kirti Chandra Sahu[2b]
[1]*Department of Chemical Engineering, Indian Institute of Technology Kanpur, Uttar Pradesh, 208016, India*
[2]*Department of Chemical Engineering, Indian Institute of Technology Hyderabad, Sangareddy, Telangana, 502 284, India*
(Dated: October 11, 2021)

I. DERIVATION OF WRIBL MODEL

A. Dimensionless governing equations

The governing equations are non-dimensionsionalised using Eq. (13) of the main manuscript. The dimensionless governing equations, by retaining all the terms of $\mathcal{O}(\delta)$ and neglecting higher order terms, are given by

$$\frac{\partial u}{\partial x} + \frac{\partial v}{\partial y} + \frac{\partial w}{\partial z} = 0, \tag{1}$$

$$\delta Re\left(\frac{\partial u}{\partial t} + u\frac{\partial u}{\partial x} + v\frac{\partial u}{\partial y} + w\frac{\partial u}{\partial z}\right) = -\delta\frac{\partial p}{\partial x} + \frac{\partial^2 u}{\partial z^2}, \tag{2}$$

$$\delta Re\left(\frac{\partial v}{\partial t} + u\frac{\partial v}{\partial x} + v\frac{\partial v}{\partial y} + w\frac{\partial v}{\partial z}\right) = -\delta\frac{\partial p}{\partial y} + \frac{\partial^2 v}{\partial z^2}, \tag{3}$$

$$\frac{\partial p}{\partial z} = 0, \tag{4}$$

where u, v, w and p are the velocity components in the x, y, z directions, and pressure field, respectively. The rest of the terms are defined in the main manuscript. Similarly, the dimensionless boundary conditions are

$$\frac{\partial u}{\partial z}(h) = -\delta E M^{T_1}, \text{ where } M^{T_1} = \frac{\partial \phi}{\partial z}\frac{\partial h}{\partial x} + \frac{\partial \phi}{\partial x}, \tag{5}$$

$$\frac{\partial v}{\partial z}(h) = -\delta E M^{T_2}, \text{ where } M^{T_2} = \frac{\partial \phi}{\partial z}\frac{\partial h}{\partial y} + \frac{\partial \phi}{\partial y}. \tag{6}$$

The terms M^{T_1} and M^{T_2} represents the non dimensional tangential Maxwell stress in x and y directions, respectively. The dimensionless normal stress balance at the interface is given by

$$p(h) = \frac{E}{2}\left[\varepsilon\left(\frac{\partial \phi_1}{\partial z}\right)^2 - \left(\frac{\partial \phi_2}{\partial z}\right)^2\right] - \frac{\delta^2}{Ca}\left[\frac{\partial^2 h}{\partial x^2} + \frac{\partial^2 h}{\partial y^2}\right] - S\left[\frac{h_f^3}{h^3} - \frac{h_f^2}{h^2}\right]. \tag{7}$$

The various dimensionless numbers are defined in the main manuscript. Now integrating the continuity equation (1) and using the kinematic condition, we obtain the evolution equation for the interface position (h) as

$$\frac{\partial q_x}{\partial x} + \frac{\partial q_y}{\partial y} + \frac{\partial h}{\partial t} = 0, \tag{8}$$

where the flow rate in the x and y directions are given by

$$q_x = \int_0^h u dz \text{ and } q_y = \int_0^h v dz, \text{ respectively.} \tag{9}$$

Integrating Eqs. (1)-(4) and eliminating pressure, we obtain

$$\delta Re\left(\frac{\partial u}{\partial t} + u\frac{\partial u}{\partial x} + v\frac{\partial u}{\partial y} + w\frac{\partial u}{\partial z}\right)dz = -\delta E M^{N_1} + \frac{\delta^3}{Ca}\frac{\partial}{\partial x}\left[\frac{\partial^2 h}{\partial x^2} + \frac{\partial^2 h}{\partial y^2}\right] + S\frac{\partial}{\partial x}\left(\frac{h_f^3}{h^3} - \frac{h_f^2}{h^2}\right) + \frac{\partial^2 u}{\partial z^2}, \tag{10}$$

---

[a] dipinsp@iitk.ac.in
[b] ksahu@che.iith.ac.in

$$\delta Re\left(\frac{\partial v}{\partial t}+u\frac{\partial v}{\partial x}+v\frac{\partial v}{\partial y}+w\frac{\partial v}{\partial z}\right)dz=-\delta EM^{N_2}+\frac{\delta^3}{Ca}\frac{\partial}{\partial y}\left[\frac{\partial^2 h}{\partial x^2}+\frac{\partial^2 h}{\partial y^2}\right]+S\frac{\partial}{\partial y}\left(\frac{h_f^3}{h^3}-\frac{h_f^2}{h^3}\right)+\frac{\partial^2 v}{\partial z^2}, \tag{11}$$

where, M^{N_1}, M^{N_2} are the non-dimensional normal component of the Maxwell stress in x and y directions, respectively, which are given by

$$M^{N_1}=\frac{1}{2}\frac{\partial}{\partial x}\left[\epsilon\left(\frac{\partial\phi_1}{\partial z}\right)^2-\left(\frac{\partial\phi_2}{\partial z}\right)^2\right]\text{and } M^{N_2}=\frac{1}{2}\frac{\partial}{\partial y}\left[\epsilon\left(\frac{\partial\phi_1}{\partial z}\right)^2-\left(\frac{\partial\phi_2}{\partial z}\right)^2\right].$$

B. WRIBL Model

We first decompose the velocity components u and v as follows:

$$u(x,y,z,t)=\hat{u}(x,y,z,t)+\tilde{u}(x,y,z,t), \tag{12}$$
$$v(x,y,z,t)=\hat{v}(x,y,z,t)+\tilde{v}(x,y,z,t), \tag{13}$$

where, $\hat{u}$ and $\hat{v}$ are the $\mathcal{O}(1)$ contributions and $\tilde{u}$ and $\tilde{v}$ denote their corresponding $\mathcal{O}(\delta)$ corrections. The leading order velocities are chosen to be locally parabolic at their respective coordinate position. This assumption is valid up to moderate Reynolds numbers. The following equations are used to find $\hat{u}$ and $\hat{v}$ as,

$$\frac{\partial^2\hat{u}}{\partial z^2}=K_u,\ \hat{u}\mid_0=0,\frac{\partial\hat{u}}{\partial z}\mid_h=0,\ q_x=\int_0^h u dz, \tag{14}$$
$$\frac{\partial^2\hat{v}}{\partial z^2}=K_v,\ \hat{v}\mid_0=0,\frac{\partial\hat{v}}{\partial z}\mid_h=0,\ q_y=\int_0^h v dz. \tag{15}$$

The terms K_u and K_v are introduced so that the leading order velocities profile is locally parabolic, and is obtained in terms of the flow rates, q_x and q_y using the integral constraint in Eqs.(14) and (15). From Eq. (1), w is given by

$$w=-\int_0^z\frac{\partial u}{\partial x}dz-\int_0^z\frac{\partial v}{\partial y}dz. \tag{16}$$

Next, we substitute the expressions for velocities obtained from Eqs. (14), (15) and (16) in Eqs. (10) and (11), and neglect all terms of $\mathcal{O}(\delta\tilde{u})=\mathcal{O}(\delta^2)$, $\mathcal{O}(\delta\tilde{v})=\mathcal{O}(\delta^2)$ or smaller. We take integral with a suitable weight function using the Galerkin method. The weight function, F can be defined as:

$$\frac{\partial^2 F}{\partial z^2}=1,\ F\mid_0=0,\ \frac{\partial F}{\partial z}\mid_h=0. \tag{17}$$

From the above, we obtain the weight function, $F=z^2/2-zh(x,t)$. After taking the weighted integral of diffusion terms in Eqs. (2),(3) and using the weight function given in Eq. (17), we get

$$\int_0^h F\frac{\partial^2\hat{u}}{\partial z^2}dz=F\frac{\partial\hat{u}}{\partial z}\mid_h+q_x, \tag{18}$$

$$\int_0^h F\frac{\partial^2\hat{v}}{\partial z^2}dz=F\frac{\partial\hat{v}}{\partial z}\mid_h+q_y. \tag{19}$$

The expression for $\frac{\partial \hat{u}}{\partial z}, \frac{\partial \hat{v}}{\partial z}$ in Eqs. (18) and (19) can be obtained using Eqs. (5) and (6). Then, the final WRIBL equations are given by

$$\frac{\partial h}{\partial t} + \frac{\partial q_x}{\partial x} + \frac{\partial q_y}{\partial y} = 0, \tag{20}$$

$$\int_0^h \delta Re F \left(\frac{\partial \hat{u}}{\partial t} + \hat{u}\frac{\partial \hat{u}}{\partial x} + \hat{v}\frac{\partial \hat{u}}{\partial y} + \hat{w}\frac{\partial \hat{u}}{\partial z} \right) dz = q_x - \delta E M^{T_1} F \,|_h + \left[\frac{\delta^3}{Ca}\frac{\partial}{\partial x}\left(\frac{\partial^2 h}{\partial x^2} + \frac{\partial^2 h}{\partial y^2} \right) - \delta E M^{N_1} + S\frac{\partial}{\partial x}\left(\frac{h_f^3}{h^3} - \frac{h_f^2}{h^2} \right) \right] \int_0^h F dz, \tag{21}$$

$$\int_0^h \delta Re F \left(\frac{\partial \hat{v}}{\partial t} + \hat{u}\frac{\partial \hat{v}}{\partial x} + \hat{v}\frac{\partial \hat{v}}{\partial y} + \hat{w}\frac{\partial \hat{v}}{\partial z} \right) dz = q_y - \delta E M^{T_2} F \,|_h + \left[\frac{\delta^3}{Ca}\frac{\partial}{\partial y}\left(\frac{\partial^2 h}{\partial x^2} + \frac{\partial^2 h}{\partial y^2} \right) - \delta E M^{N_2} + S\frac{\partial}{\partial y}\left(\frac{h_f^3}{h^3} - \frac{h_f^2}{h^2} \right) \right] \int_0^h F dz, \tag{22}$$

$$\delta O_c \left(\frac{\partial Q}{\partial t} + \hat{u}\frac{\partial Q}{\partial x} + \hat{v}\frac{\partial Q}{\partial y} + Q\frac{\partial h}{\partial x}\frac{\partial \hat{u}}{\partial z} + Q\frac{\partial h}{\partial y}\frac{\partial \hat{v}}{\partial z} - Q\frac{\partial \hat{w}}{\partial z} \right) = \left(\frac{\partial \phi_2}{\partial z} - \sigma\frac{\partial \phi_1}{\partial z} \right). \tag{23}$$

These equations are the same as Eqs. (14)-(17) in the main manuscript. In the 2D configuration, we recover the governing equations given in Pillai *et al.* [1].

II. GRID CONVERGENCE TEST

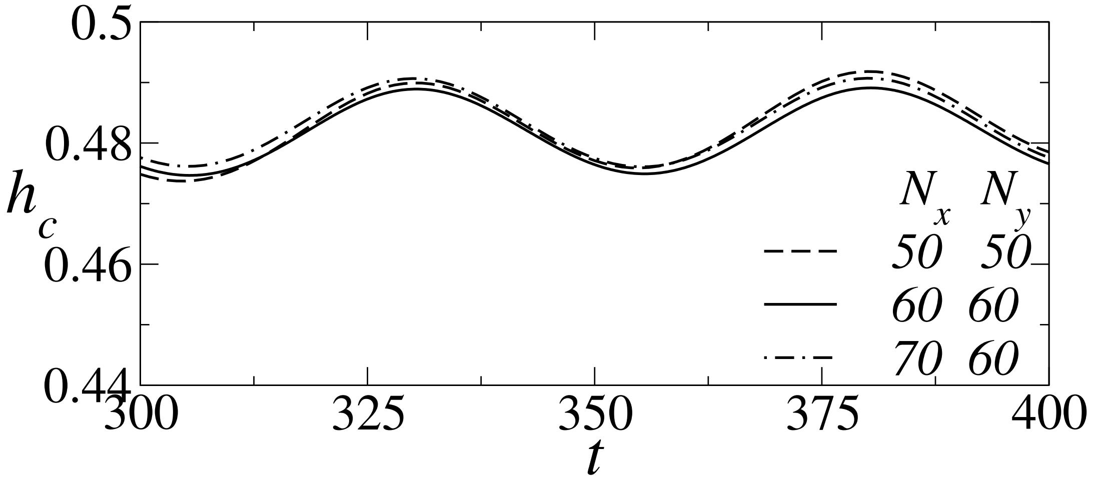

Supplementary Figure 1: Temporal variation of droplet height, h_c (in the quasi-steady region) obtained using different grids under AC forcing. The parameters are $Re = 1$, $S = 20$, $\varepsilon = 10$ and $\sigma = 10$, $E_{AC} = 10$ and $\Omega = \pi/50$. The maximum error between $N_x \times N_y = (60 \times 60)$ and (70×60) is less than 2%. This error is found to be less than 1% in the corresponding DC case.

III. COMPARISON BETWEEN 2D AND 3D CONFIGURATIONS

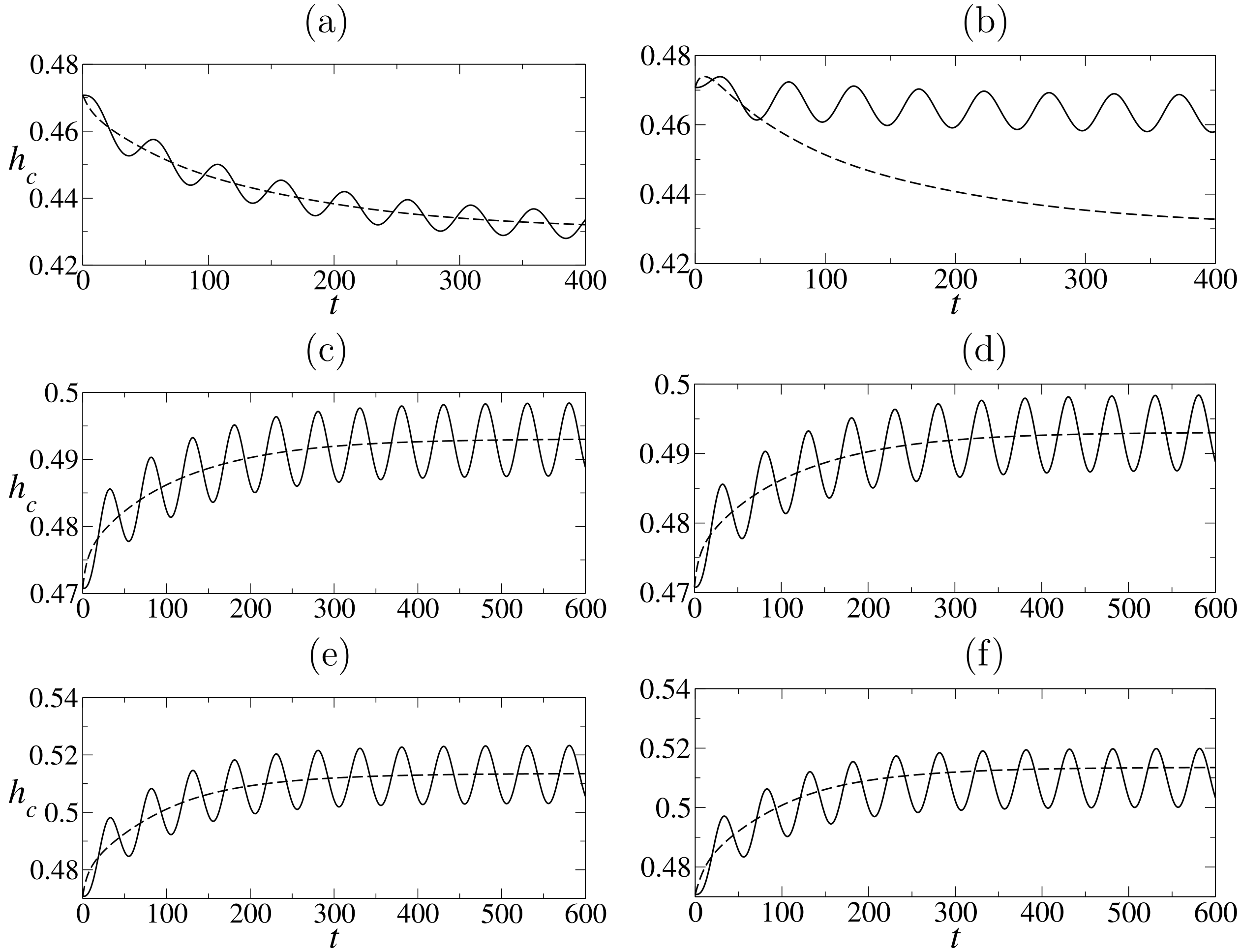

Supplementary Figure 2: Temporal variation of droplet height, h_c for $Re = 1$, $S = 20$ obtained using two-dimensional simulations: (a,c,e) $O_c = 0.01$ and (b,d,f) $O_c = 10$. Panels (a,b), (c,d) and (e,f) are for ($\varepsilon = 10$ and $\sigma = 6$), ($\varepsilon = 10$ and $\sigma = 10$) and ($\varepsilon = 6$ and $\sigma = 10$), respectively. Here, for all the AC cases, $E_{AC} = 10$ and $\Omega = \pi/50$, and for all the DC cases, $E_{DC} = 5$.

All 2D results are obtained with 200 grid points, as detailed in Pillai *et al.* [1].

[1] D. S. Pillai, K. C. Sahu, and R. Narayanan, Electrowetting of a leaky dielectric droplet under a time-periodic electric field, Phys. Rev. Fluids **6**, 073701 (2021).
